# Prognostic Value of Clinical Tests in Neonates With Hypoxic-Ischemic Encephalopathy Treated With Therapeutic Hypothermia: A Systematic Review and Meta-Analysis

**DOI:** 10.3389/fneur.2020.00133

**Published:** 2020-02-25

**Authors:** Weiqin Liu, Qifen Yang, Hong Wei, Wenhui Dong, Ying Fan, Ziyu Hua

**Affiliations:** ^1^Department of Neonatology, Ministry of Education Key Laboratory of Child Development and Disorders, National Clinical Research Center for Child Health and Disorders, China International Science and Technology Cooperation Base of Child Development and Critical Disorders, Children's Hospital of Chongqing Medical University, Chongqing, China; ^2^Chongqing Key Laboratory of Pediatrics, Children's Hospital of Chongqing Medical University, Chongqing, China; ^3^School of Life Sciences, SouthWest University, Chongqing, China

**Keywords:** therapeutic hypothermia, hypoxic-ischemic encephalopathy, neonates, clinical test, prognosis

## Abstract

**Background and Objective:** There remains an unmet clinical need for markers that predict outcomes in the hypothermia-treated (HT) infants with HIE. The aim of this meta-analysis was to investigate the prognostic accuracy of currently available clinical tests performed in the immediate post-natal period for predicting neurological outcomes between 18 months and 3 years of age in HT near-term and term infants with perinatal asphyxia and HIE.

**Methods:** A comprehensive review of the Embase, Cochrane library, and PubMed databases was performed to identify studies that evaluated the prognostic value of clinical tests for neurological outcomes in HT near-term and term infants with perinatal asphyxia and hypoxic-ischemic encephalopathy. Pooled sensitivity and specificity with corresponding 95% confidence intervals and area under the receiver operating characteristic (ROC) curve (AUC) were calculated.

**Results:** Of the 1,144 relevant studies, 26 studies describing four clinical tests conducted in 1458 HT near-term or term infants were included. For predicting an unfavorable neurological outcome, of the imaging techniques, MRI within 2 weeks of birth performed best on sensitivity 0.85 (95% *CI* 0.79–0.89), specificity 0.72 (95% *CI* 0.66–0.77), and AUC 0.88; among the neurophysiological tests, multichannel EEG (Electroencephalogram) demonstrated the sensitivity 0.63 (95% *CI* 0.49–0.76), specificity 0.82 (95% *CI* 0.70–0.91), and AUC 0.88, and for aEEG (amplitude-integrated electroencephalography) background pattern pooled sensitivity, specificity and AUC were 0.90 (95% *CI* 0.86–0.94), 0.46 (95% *CI* 0.42–0.51), and 0.78 whereas for SEPs (Somatosensory evoked potentials), pooled sensitivity and specificity were 0.52 (95% *CI* 0.34–0.69), 0.76 (95% *CI* 0.63–0.87), and AUC 0.84, respectively.

**Conclusions:** In the wake of the era of TH, MRI and neurophysiological tests (aEEG or EEG) were promising predictors of adverse outcomes, while SEPs need high-quality studies to confirm the findings. Continued follow-up of the children and well-designed large prospective studies are essential to determine whether these benefits are maintained in later childhood.

## Introduction

Hypoxic-ischemic encephalopathy (HIE) after perinatal asphyxia is the primary cause of death or long-term neurological impairment in infants born at term. Early predictive indicators of neurological outcomes in infants with HIE is essential for making rational clinical decisions. Before the era of therapeutic hypothermia, the prognostic value of MRI (first week) in neonates with HIE has been well-validated. aEEG or EEG recorded within the first 7 days of life in term infants may have potential as a neurophysiologic predicting tests. However, the prognostic value of SEP should be interpreted with caution due to small sample sizes (1).

Therapeutic hypothermia for 72 h provides neuroprotection that significantly improves survival and neurological outcomes in term infants with moderate to severe HIE (2). However, the prognostic capability of these parameters may vary under hypothermic conditions, and there remains an unmet clinical need for markers that predict outcomes in hypothermia-treated (HT) infants (3, 4).

To the authors' knowledge, there are no published meta-analyses that investigate the prognostic capabilities of currently available clinical tests for predicting long-term neurological outcomes in HT term-infants with HIE. Therefore, a meta-analysis was conducted to evaluate the prognostic accuracy of currently available clinical tests performed in the immediate post-natal period for predicting neurological outcomes between 18 months and 3 years of age in HT near-term and term infants with perinatal asphyxia and HIE.

## Methods

### Search Strtegy

Three review authors (W.L, Q.Y and Z.H) independently searched the PubMed, Cochrane Library, and Embase databases from 2009 to February 2019 using the following keywords “hypoxic-ischemic encephalopathy” AND “newborn” AND “hypothermia”; “neonatal Encephalopathy” AND “newborn” AND “hypothermia” Searches were limited to literature published in the English language.

### Inclusion and Exclusion Criteria

Inclusion criteria were: (1)observational prognostic studies that included infants ≥35 weeks of gestation with perinatal asphyxia and HIE diagnosed according to clearly described criteria; (2) that were treated with therapeutic hypothermia; and (3) that underwent neurological follow-up longitudinally until ≥18 months of age, with outcomes defined as good or adverse.

Exclusion criteria were: (1) reviews, letters, abstracts, or editorials; or (2) studies that reported insufficient data, (3) Non-English language, (4) Uncertain follow-up time and Follow-up time is >18 months.

### Study Selection

Three review authors (W.L, Q.Y, and Z.H) independently screened the titles and abstracts identified by the search for potentially relevant studies. Texts were retrieved and reviewed to determine which studies met the inclusion criteria. Disagreements about data extraction were resolved by discussion with a third review author (ZH) until consensus was reached.

### Data Extraction

Three review authors (W.D, H.W., and Y.F) independently extracted data from the included studies using a data extraction sheet. The following information was recorded: authors, year of publication, number of study subjects, gestational age, birth weight, Apgar score, blood pH, clinical tests (e.g., imaging, neurophysiological, other), neurological tests, and length of follow-up. If both hypothermic and normothermic infants were included in a study, data about the hypothermic infants were extracted separately. According to the review of the studies, we established the MRI subgroup and the aEEG subgroup.

### Statistical Analysis

Statistical analyses were conducted with *RevMan v*5.3 and *Meta-Disc* 1.4. Three review authors (W.L, Q.Y, and Z.H) examined the quality of the included studies using *Quadas* 2, which evaluates four key domains, including patient selection, index test, reference standard, and flow and timing and independently extracted individual patient data from each of the studies into a predefined database. Personal patient data from all studies were pooled to create 2 × 2 tables and pooled sensitivity and specificity with 95% confidence intervals (*CI*s) and area under the receiver operating characteristic curve (AUC) were calculated. Heterogeneity between studies was tested with the inconsistency (*I*^2^) index and χ^2^- test. A fixed-effect model was used if there was no evidence of heterogeneity between studies (*I*^2^ <50%, *P* > 0.05); otherwise, a random-effects model was used.

## Results

### Study Identification

**Figure 1:**
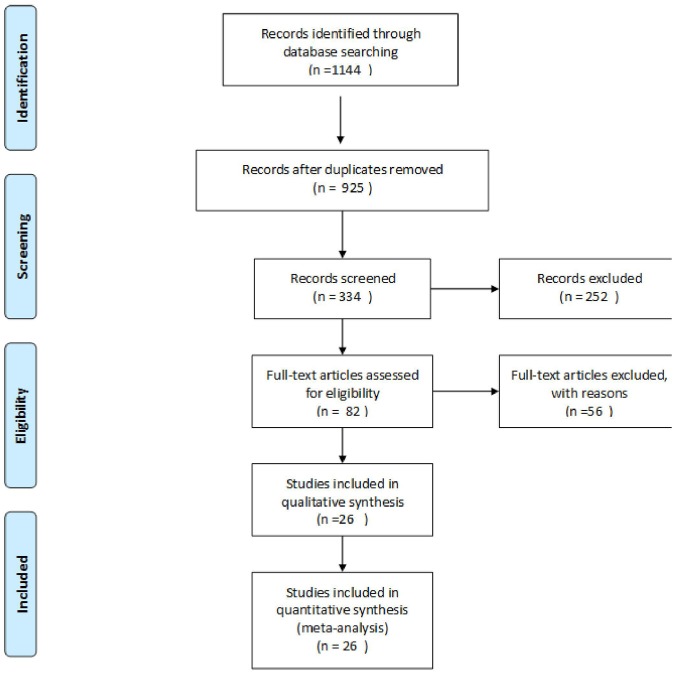
Flowchart of the search and selection process.

The searches identified 1,144 articles. Following the removal of duplicates, the titles and abstracts of 925 studies were screened, and 82 studies were considered potentially eligible for inclusion in this meta-analysis. Full-text articles were retrieved and reviewed; among these, 11 studies were excluded because they were not published in the English language, 27 studies were excluded due to missing data. Fifteen studies were excluded due to uncertain follow-up time and follow-up time is <18 months, and three studies were not analyzed due to small sample size. Finally, 26 reviews were included in the pooled analyses (Figure 1 and Supplementary Table 1).

### Characteristics of Included Studies

The characteristics of the included studies are shown in Tables 1, 2. The 26 eligible studies included 1458 HT near-term or term infants with perinatal asphyxia and HIE and follow up available after 18 months of age. Clinical tests performed in the immediate postnatal period included brain magnetic resonance imaging (MRI), EEG, aEEG, SEPs. Neurological examinations were performed between 18 months and 3 years of age.

**Table 1:** Characteristics of included studies.

**References**	**Time**	**Study design**	**Number of patients** **(Female/** **Male Ratio)**	**Gestational age (W)**	**Birth weight (Kg)**	**Apgar score (1/5/10 min)**	**Blood pH**
Rutherford et al. (5)	2010	P	64 (25/39)	40(39–41)	3.45(2.995–3.863)	4 (3–5) (10min)	Unknown
Thoresen et al. (6)	2010	P	43 (20/23)	40	3.38 (0.80)	5 (3.75) (10 min)	6.95 (0.16)
Shankaran et al. (7)	2011	P	57 (Unknown)	≥36	Unknown	Unknown	6.9 (0.2)
Takenouchi et al. (8)	2011	P	29 (Unknown)	Poor outcome 38 (1.7) Good outcome 39 (1.5)	Poor outcome 3.350 (0.767) Good outcome 3.234 (0.559)	Unknown	Poor outcome 6.91 (0.21) Good outcome 6.84 (0.26)
Shankaran et al. (9)	2012	P	73 (37/36)	39.1 (1.5)	3.328 (0.557)	Unknown	6.9 (0.2)
Cseko et al. (10)	2013	R	70 (28/42)	Poor outcome 39 (37–40) Good outcome 39 (37.5–40)	Poor outcome 3.26 (2.95–3.488) Good outcome 3.3 (2.948–3.558)	Poor outcome 3 (2–4) (5 min) Good outcome 5 (3–6.25) (5 min)	Poor outcome 6.89 (6.82–7.1) Good outcome 7.2 (7.02–7.27)
Lemmers et al. (11)	2013	P	39 (15/24)	Good outcome 40.32 (1.5) Poor outcome 40.21 (1.26)	Good outcome 3.744 (0.644) Poor outcome 3.831 (0.603)	Good outcome 3 (1–6) (5 min) Poor outcome 3 (0–7) (5 min)	Good outcome 6.97 (0.2) Poor outcome 6.90 (0.3)
Li et al. (12)	2013	R	21 (13/8)	Good outcome 38.8 (1.9) Poor outcome 39.2 (1.9)	Good outcome 3.071 (0.507) Poor outcome 2.867 (0.470)	Good outcome 4.2 (2.7) (5 min) Poor outcome 3.5 (2.2) (5 min)	Good outcome 7.07 (0.22) Poor outcome 7.13 (0.26)
Chalak et al. (13)	2014	P	90 (35/55)	39 ± 2	3.43 (0.584)	2 (0–7) (1 min) 4 (0–9) (5 min)	6.97 (0.17)
Azzopardi (14)	2014	P	147 (Unknown)	40 (39–41)	Unknown	3 (1–5) (5 min)	6.9 (6.78–7.01)
Del Balzo et al. (15)	2014	P	20 (Unknown)	≥36	≥1.80	Unknown	Unknown
Garfinkle et al. (16)	2015	R	26 (10/16)	38.8	3.336 (0.606)	4 (3–5) (5 min)	6.96 (0.13)
Alderliesten et al. (17)	2015	P	65 (Unknown)	40.1 (1.6)	3.239 (0.469)	2 (3) (1 min) 5 (2) (5 min)	6.99 (0.18)
Charon et al. (18)	2016	R	43 (Unknown)	Good outcome 39+5 (38+2–40+6) Poor outcome 39+2 (39+4–41)	Good outcome 3.17 (2.775–3.460) Poor outcome 3.6 (3.07–3.872)	Good outcome 4 (3–5) (5 min) Poor outcome 3 (1.5–4.5) (5 min)	Good outcome 7.26 (7.08–7.33) Poor outcome 7.25 (7.12–7.30)
Weeke et al. (19)	2016	P	26 (11/15)	40.4	3.445 (2.261–4.75)	4 (1–10) (5 min)	6.89 (0.20)
Chiang et al. (20)	2016	P	12 (4/8)	Good outcome 39.5 (0.6) Poor outcome 37.9 (1.5)	Good outcome 2.658 (0.465) Poor outcome 3.525 (0.828)	Good outcome 4 (1; 5) (5 min) Poor outcome 4 (3; 7) (5 min)	Good outcome 6.97 (6.73–7.32) Poor outcome 7.02 (6.72–7.43)
Heursen et al. (21)	2017	P	54 (24/30)	39.4 (1.64)	3.323 ± 0.527	2 (0–6) (1 min) 4.5 (0–8) (5 min)	6.93 (0.13)
Ahearne et al. (22)	2017	P	33 (11/22)	Good outcome 40.4 (39.2–41.1) Poor Outcome 40.8 (39.7–41.4)	Good outcome 3.5 (3.2–4.1) Poor Outcome 3.4 (3.1–3.7)	Unknown	Unknown
Cainelli et al. (23)	2018	P	35 (Unknown)	38 (37; 40)	3.280 (2.665; 3.515)	3 (1; 5) (1 min) 5 (4; 6) (5 min) 7 (5; 8) (10 min)	7.0 (6.8; 7.1)
Nevalainen et al. (24)	2017	R	24 (13/11)	39.6 (1.5)	3.350 (0.600)	1 (1 min) 2 (5 min) 3.5 (10 min)	6.9 (0.2)
Trivedi et al. (25)	2017	P	57 (29/28)	38.5 (1.6)	3.166 (0.688)	4 (5 min) 5 (10 min)	7.05 (0.19)
Skranes et al. (26)	2017	P	47 (Unknown)	≥36 weeks	Unknown	Unknown	Unknown
Weeke et al. (27)	2017	P	122 (53/69)	40.0 (2.2)	3.50 (0.858)	3 (5 min)	6.90 (0.25)
Liu et al. (28)	2017	P	165 (62/103)	≥36 weeks	Unknown	Unknown	Unknown
Barta et al. (29)	2018	R	51 (20/31)	Good outcome 39 (38;40) Poor outcome 38 (37;40)	Good outcome 3.261 (0.577) Poor outcome 3.128 (0.537)	Good outcome 5 (4–7) (5 min) Poor outcome 3 (2–4) (5 min)	Good outcome 7.21 (6.98–7.28) (1 h) Poor outcome 7.10 (7.00–7.20) (1 h)
De Wispelaere et al. (30)	2019	R	45 (23/22)	39+6 (38+1–40+4)	3.29 ± 0.612	3 (1–4) (5 min)	6.96 ± 0.24

*Prospective study (P) Retrospective study (R). Values represent the mean (SD), median [IQR], or median (range). unless otherwise indicated*.

**Table 2:** Neurological outcomes defined by study.

**References**	**Time**	**Index** **test**	**Abnormal findings**	**Follow up** **(m)**	**Neurodevelopmental assessment** **(definition of adverse outcome)**
Rutherford et al. (5)	2010	MRI	Abnormal signal in the WM, BGT, PLIC, COR, or various combinations of such lesions	18	Bayley II and GMFCS; death or disability (MDI <70, GMFCS level 3–5, or altered vision or hearing)
Thoresen et al. (6)	2010	aEEG	Lower margin ≤ 5 μV and upper margin>10 μV or <10 μV (voltage classifications) BS, LV, and FT traces (pattern classification)	18	Bayley II and GMFCS; death or disability (MDI <70, GMFCS level 3–5, or altered vision or hearing)
Shankaran et al. (7)	2011	aEEG	BS, LV, and FT traces (pattern classification)	18	Bayley II and GMFCS; death or disability (MDI <85, GMFCS level 2–5, or altered vision or hearing)
Takenouchi et al. (8)	2011	MRI	Abnormal signal in the BGT, severe extensive supratentorial restricted diffusion.	18	Bayley III; death or severe disability (MDI <70 or severe motor deficit restricting
Shankaran et al. (9)	2012	MRI	abnormal signal in the WM, BGT, PLIC, ALIC, COR, or various combinations of such lesions (NICHD brain injury pattern scale)	18–22	Bayley II and GMFCS; death or disability (MDI <85, GMFCS level 2–5, or altered vision or hearing, persistent seizure disorder)
Cseko et al. (10)	2013	aEEG	BS, LV, and FT traces	18–24	Bayley II; death or disability (MDI or PDI <85)
Lemmers et al. (11)	2013	aEEG	BS, LV, and FT traces	18	Griffiths and neurologic examination: Death or DQ <85
Li et al. (12)	2013	MRI EEG	Abnormal signal in the WM, BGT, COR, or various combinations of such lesions Background EEG depression (classification of Watanabe)	24	K-Form Developmental Test: death, CP, hearing impairment, or blindness, DQ <70
Chalak et al. (13)	2014	MRI	Abnormal signal in the WM, BGT, PLIC, ALIC, COR, or various combinations of such lesions (NICHD brain injury pattern scale)	24	Bayley III; Death, cerebral palsy, Bayley scores >1 SDs from the norm, Bayley <85
Azzopardi (14)	2014	aEEG	Lower margin ≤ 5 μV and upper margin >10 μV or <10 μV (voltage classifications) BS, LV, and FT traces (pattern classification)	18	Bayley II and GMFCS; death or disability (MDI <70, GMFCS level 3–5, or altered vision or hearing)
Del Balzo et al. (15)	2014	EEG MRI	Classification of EEG background activity (Pressler et al. score) Abnormal signal in the WM, BGT, PLIC, ALIC, COR, or various combinations of such lesions	18	Bayley III; death or severe disability (cognitive development index 3 S.D.s below mean or severe sensorimotor disability)
Garfinkle et al. (16)	2015	SEPs MRI	N19 potentials absent or prolonged (unilaterally or bilaterally) Abnormal cortex and BGT, or various combinations of such lesions(Barkovich score)	24	Bayley III; death or Bayley <80, GMFCS level 2–5, or altered vision or hearing
Alderliesten et al. (17)	2015	MRI	Abnormal cortex and BGT, or various combinations of such lesions (Barkovich score)	18	GMDS; death, CP, or DQ <85
Charon et al. (18)	2016	MRI	Abnormal cortex and BGT, or various combinations of such lesions(Barkovich score)	18–41	RBL scale and GMFCS: death or disability (DQ <70 and GMFCS level 3–5)
Weeke et al. (19)	2016	MRI EEG	Abnormal cortex and BGT, or various combinations of such lesions(Barkovich score) Classification of EEG background activity (Pressler et al. score)	24	Bayley III; death, CP, hearing impairment, or blindness, Bayley <85
Chiang et al. (20)	2016	MRI	Abnormal signal in the WM, BGT, PLIC, or various combinations of such lesions	24	Bayley III; disability(CP, bilateral blindness, or bilateral hearing loss) or Neurodevelopmental delay
Heursen et al. (21)	2017	MRI	Abnormal signals of the BGT or WM, or Near-total injury	24	Bayley III and GMFCS; CP, death or disability GMFCS level 2–5 or altered vision or hearing)
Nevalainen et al. (24)	2017	SEP	Bilaterally absent SEPs	18	Unfavorable outcomes included death or severe neurological sequelae comprising severe epilepsy, tetraparesis or dyskinetic cerebral palsy or severe psychomotor retardation
Trivedi et al. (25)	2017	MRI	Abnormal signals of the subcortical region, or white matter; or cortex, or cerebellum and brainstem	18	Bayley III; death or a Bayley-III score of <85 in any domain
Skranes et al. (26)	2017	aEEG	BS, LV, and FT traces (pattern classification)	24	(Bayley III and GMFCS; CP, death or cognitive or motor scores of <85, GMFCS level 3–5, or altered vision or hearing)
Ahearne et al. (22)	2017	EEG	Classification of EEG background activity (Pressler et al. score)	36–42	Bayley III; death or cognitive, language, and motor scores of <85, dyskinetic, or spastic quadriplegic cerebral palsy or autism
Weeke et al. (27)	2017	aEEG	BS, LV, and FT traces (pattern classification)	≥24	Bayley III and GMFCS; death, CP, severe hearing, or visual impairments, or an adverse neurodevelopment (Bayley score <85, Griffiths developmental quotient <88)
Liu et al. (28)	2017	aEEG	Lower margin ≤ 5 μV and upper margin >10 μV or <10 μV (voltage classifications) BS, LV, and FT traces (pattern classification)	24	Bayley III and GMFCS; Bayley score <85, GMFCS 3–5, severe visual deficits, or severe bilateral hearing loss
Barta et al. (29)	2018	MRI aEEG	Abnormal cortex and BGT, or various combinations of such lesions(Barkovich score) BS, LV, and FT traces (pattern classification)	24	Bayley III and GMFCS; death or disability (MDI or PDI <85, GMFCS level 2–5, or altered vision or hearing)
Cainelli et al. (23)	2018	EEG SEP	Classification of EEG background activity (Pressler et al. score) N20 OR N13 potentials absent or prolonged (unilaterally or bilaterally)	24	GMDS; death, CP or DQ <85
De Wispelaere et al. (30)	2019	MRI	Abnormal cortex and BGT, or various combinations of such lesions(Barkovich score)	24	Bayley III and GMFCS; death or disability (MDI or PDI <85, GMFCS level 2–5, or altered vision or hearing)

*BSID-II or BSID-III, Bayley Scales of Infant Development II or III; WPPSI, Wechsler Preschool and Primary Scale of Intelligence III; GMFCS, Gross Motor Function Classification System; GMDS, Griffiths mental development scales; RBL, Revised Brunet-Lezine scale; MDI, Mental Developmental Index; PDI, Psychomotor Developmental Index; CP, Cerebral palsy; DQ, Developmental quotient; SEPs, Somatosensory evoked potentials; EEG, Electroencephalogram; MRI, Magnetic resonance imaging; aEEG, amplitude-integrated electroencephalogram; BGT, basal ganglia and thalamus; ALIC/PLIC, anterior or posterior limb of the internal capsule; WM, white matter; COR, cortex; BS, burst-suppression; LV, low voltage; FT, FT flat trace*.

### Methodological Quality of Included Studies

**Figure 2:**
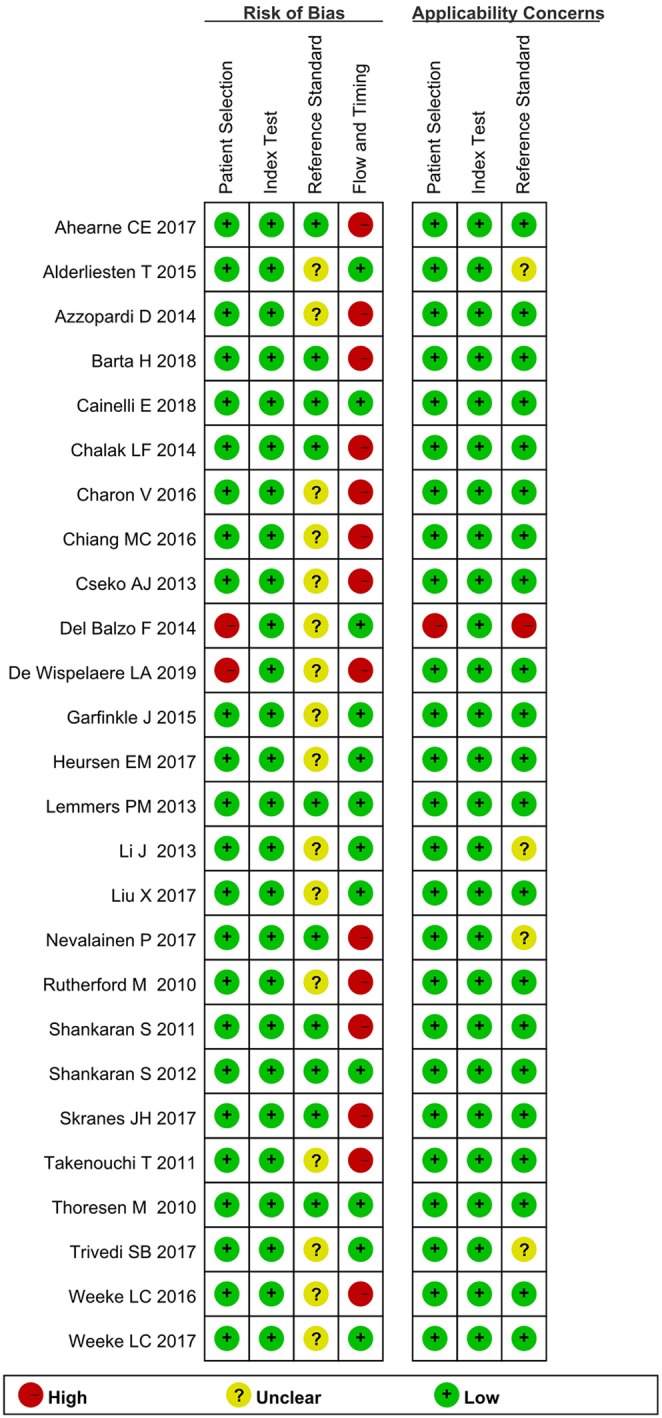
Quality assessment of included studies using QUADAS-2.

**Figure 3:**
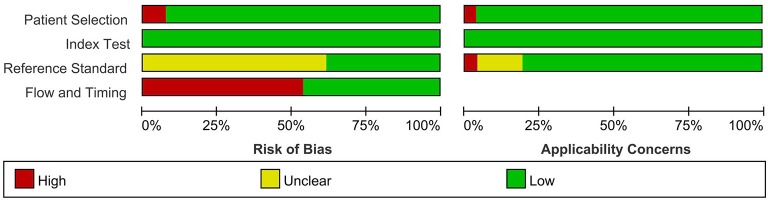
Percentage of the studies with risk of bias and applicability concerns in the different domains of QUADAS-2.

The risks of bias in the index test and patient selection were low (Figures 2, 3). Sixteen studies (5, 8, 10, 12, 14–21, 25, 27, 28, 30) did not indicate whether the reference standard results were interpreted without knowledge of the results of the index test. For flow and timing, 14 studies (5, 7, 8, 10, 13, 14, 18–20, 22, 24, 26, 29, 30) did not include all patients in the analyses. Overall, most of the included studies did not have a high risk of bias.

### Prognostic Value of Clinical Tests

**Figure 4:**
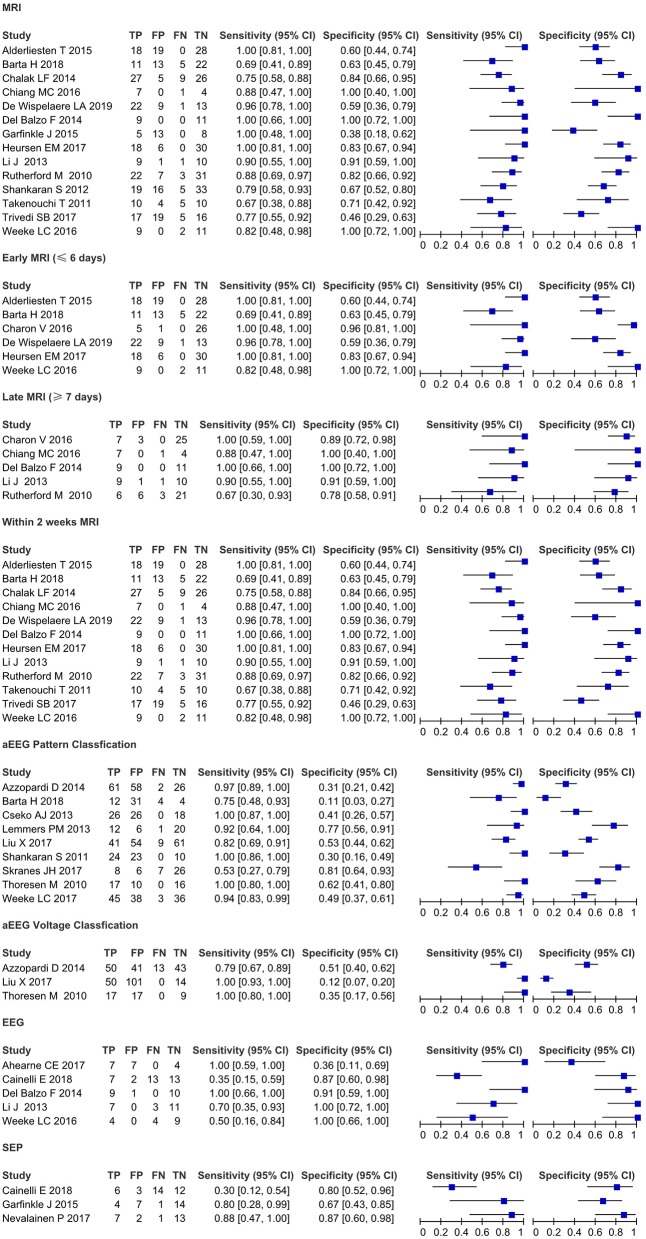
Forest plots of sensitivity and specificity as calculated from the original reports.

The results of the meta-analysis are shown in Table 3 (pooled sensitivities and specificities with confidence intervals) and Figure 4 (forest plots of sensitivity and specificity as calculated from the original reports).

**Table 3:** Pooled sensitivities and specificities with confidence intervals for tests where pooling was possible.

**Test**	**No. of studies**	**No. of patients**	**Pooled sensitivity**		**Pooled specificity**		**AUC**
			**Point Estimate**	**95% *CI***	**Point Estimate**	**95% *CI***	
MRI	14	605	0.85	0.79–0.89	0.69	0.64–0.74	0.87
Early MRI (≤ 6 days)	6	269	0.91	0.83–0.96	0.73	0.66–0.79	0.92
late MRI (≥7 days)	5	124	0.88	0.75–0.96	0.88	0.78–0.94	0.94
MRI within 2 weeks	12	506	0.85	0.79–0.89	0.72	0.66–0.77	0.88
aEEG Background classification	9	741	0.90	0.86–0.94	0.46	0.42–0.51	0.78
aEEG voltage classification	3	355	0.90	0.84–0.95	0.32	0.26–0.39	0.66
EEG	5	111	0.63	0.49–0.76	0.82	0.70–0.91	0.88
SEPs	3	84	0.52	0.34–0.69	0.76	0.63–0.87	0.84

### Imaging: Brain MRI

Fourteen studies (5, 8, 9, 12, 13, 15–17, 19–21, 25, 29, 30) evaluated the prognostic value of brain MRI(*I*^2^ = 47.9%, fixed-effect model) for neurological outcomes in HT near-term and term infants with perinatal asphyxia and HIE. Pooled sensitivity and specificity were 0.85 (95% *CI* 0.79–0.89) and 0.69 (95% *CI* 0.64–0.74), and AUC was 0.87 for predicting an unfavorable outcome. Early MRI (≤ 6 days from birth) (*I*^2^ = 60%, random-effects model) performed best on sensitivity 0.91 (95% *CI* 0.83–0.96). Late MRI (≥7 days from birth) (*I*^2^ = 33%, fixed-effect model) performed best on specificity 0.88 (95% *CI* 0.78–0.94). MRI within 2 weeks (*I*^2^ = 59.5%, random-effects model) of birth performed best on sensitivity 0.85 (95% *CI* 0.79–0.89), specificity 0.72 (95% *CI* 0.66–0.77), and AUC 0.88.

### Neurophysiological Tests: aEEG, EEG, and SEPs

Nine studies (6, 7, 10, 11, 14, 26–29) evaluated the prognostic value of aEEG tests for neurological outcomes in HT near-term and term infants with perinatal asphyxia and HIE. For aEEG background patterns (*I*^2^ = 65.2%, random-effects model), pooled sensitivity and specificity were 0.90 (95% *CI* 0.86–0.94) and 0.46 (95% *CI* 0.42–0.51), and AUC was 0.78 for predicting an unfavorable outcome. For aEEG voltage classification (*I*^2^ = 36.7, fixed-effect model), pooled sensitivity and specificity were 0.90 (95% *CI* 0.84–0.95) and 0.32 (95% *CI* 0.26–0.39), and AUC was 0.66 for predicting an unfavorable outcome.

Multichannel EEG (*I*^2^ = 17.7%, fixed-effect model) demonstrated the sensitivity 0.63 (95% *CI* 0.49–0.76), specificity 0.82 (95% *CI* 0.70–0.91), and AUC 0.88. Three studies (16, 23, 24) evaluated the prognostic value of SEPs for neurological outcomes in HT near-term and term infants with perinatal asphyxia and HIE. For SEPs (*I*^2^ =57.9%, random-effects model), pooled sensitivity and specificity were 0.52 (95% *CI* 0.34–0.69) and 0.76 (95% *CI* 0.63–0.87) for predicting an unfavorable outcome, and AUC 0.84.

## Discussion

HIE after perinatal asphyxia is a significant cause of mortality and morbidity in newborns, accounting for approximately 20% of cerebral palsy cases (31, 32). Evaluating long-term neurological outcomes based on clinical evaluations in the immediate post-natal period can be challenging. However, a reliable, evidence-based prognosis is essential for parental counseling regarding possible long-term sequelae. Hypothermia is generally accepted as the safest method for reducing neurological morbidity and mortality in infants with perinatal asphyxia and HIE (33, 34). Although the literature is replete with studies evaluating novel but relatively unknown test modalities, to ensure clinical relevance. The present review focused on tests that are widely used in clinical practice and did not attempt to provide an overview of all available procedures (1).

Current MRI injury scoring systems published in the literature include the Barkovich, the National Institutes for Child Health and Development (NICHD) and Rutherford systems (5, 9, 35). Each scoring system has some limitations. The previous does not explicitly address posterior limb of the internal capsule injury, whereas the latter two systems do not include diffusion-weighted imaging (DWI) (25), and Clinicians often evaluate the neonatal brain MRI as a whole test rather than specific brain MRI components. So, we considered parenchymal (gray or white matter) abnormalities on T1, T2, and diffuse weighted images to define an abnormal MRI. In the systematic review, brain MRI predicted an unfavorable neurological outcome in HT infants with perinatal asphyxia and HIE with a sensitivity of 0.85. Accordingly, in the TOBY trial, the accuracy of prediction of death or disability to 18 months of age by MRI was 0.84 (0.74–0.94) in HT infants and 0.81 (0.71–0.91) in a normothermic group (5). Evidence suggests that therapeutic hypothermia without affecting the overall predictive value of MRI as a marker of neurological impairment (5, 13).

In the era of predates hypothermia treatment, late MRI (8–30 days) had higher sensitivity but lower specificity than early MRI (1–7 days) (36). However, the current literature does not provide detailed individual data on the time of neonatal brain MRI in HT (37). In the present review, late MRI predicted an unfavorable neurological outcome with a sensitivity and specificity of 0.88 and 0.88, respectively, whereas early MRI showed less specificity of 0.73. However, Charon et al. reported that the specificity of MRI for predicting death or disability to 18 months of age in HT infants with HIE was 96.3% in the first week and 89.3% in the second week (18). The discrepancy between our findings and those reported by Charon et al. might be explained by the various abnormal findings and the thresholds for the index tests. Results from the present meta-analysis indicate that within 2 weeks of birth correctly predict neurological outcomes at 18 months of age in HT infants with HIE (pooled sensitivity, 0.85 [95% *CI*, 0.79–0.89].

Deep gray matter lactate/N-acetyl aspartate (Lac/NAA) peak/area ratio is the most quantitative biomarker for prediction of neurodevelopmental outcomes in magnetic resonance spectroscopy (38). However, the equipment is not widely used in clinical practice, and based on limited available studies, the validity of the results has not been quantified in previous reviews of the literature.

aEEG is the commonly used neurophysiological tests for assessment of HIE severity, for monitoring improvement over time, and for predicting neurological outcomes in infants (39). aEEG can be performed at the bedside, and background patterns and voltage classification have been considered an early predictor of neurological outcomes in HT infants with HIE. In normothermic infants, a persistently abnormal aEEG between 6 and 24 h of age is considered predictive of adverse outcomes (6, 40). However, the predictive value of aEEG for subsequent neurological impairment is altered by hypothermia. In one study, the positive prognostic value of an abnormal aEEG increased from 6 to 48 h of age in HT term infants with HIE. This shift in prognostic accuracy may be explained, at least in part, by the neuroprotective effects of therapeutic hypothermia (41). In the present review, aEEG background pattern predicted an unfavorable neurological outcome with a sensitivity and specificity of 0.90 and 0.46, respectively, and an AUC of 0.78, while aEEG voltage classification was less predictive [sensitivity 0.90 [95% *CI* 0.84–0.95]; specificity 0.32 [95% *CI* 0.26–0.39], AUC 0.66]. Possible explanations are that the sedative drugs and anticonvulsants that are commonly administered to HT infants have prolonged half-lives and increased plasma levels compared to normothermic conditions, causing voltage fluctuations on the EEG signal (12). In accordance with our findings, Shany et al. reported that background pattern was more sensitive than voltage classification for predicting neurological outcomes in infants with HIE, although assessment of background pattern may be more subjective (42).

Multichannel EEG is the gold standard for assessment of the severity of HIE and for monitoring improvement over time (39). In our review, EEG predicted subsequent neurological impairment in HT infants with HIE with a sensitivity of 0.63 (95% *CI*, 0.49–0.76), specificity of 0.82 (95% *CI*, 0.70–0.91), and an AUC of 0.88, yielding a higher specificity than aEEG. However, EEG is a relatively complex technique. Technicians are required to site EEG leads and specialists are needed to interpret neurophysiology, but these resources may not be readily available (39). High seizure burden and sleep-wake cycling have been independently associated with poor outcomes in HT infants with HIE (41). Unfortunately, lack of data meant that these parameters could not be examined in the present meta-analysis.

SEPs assess the deep brain structures such as the thalami and brainstem, which are known to be selectively vulnerable to hypoxia and ischemia (43). Several studies had shown that normal SEPs were strongly predictive of a favorable outcome, and absent SEPs were strongly predictive of an unfavorable outcome (44, 45). In our review, SEPs predicted an unfavorable neurological outcome in HT infants with HIE with a sensitivity of 0.52 (95% *CI*, 0.34–0.69) and a specificity of 0.76 (95% *CI*, 0.63–0.87). Although SEPs may provide complementary prognostic resources to HIE after perinatal asphyxia, the predictive value of SEPs investigated in this review should be interpreted with caution due to small sample sizes.

Although not explored in this review, we propose that a combination of the above prognostic tests performed would provide the greater prognostic accuracy for predicting long-term neurological outcomes in the HIE infants undergoing HT.

### Strengths and Weaknesses

To the authors' knowledge, this is the first meta-analysis to investigate the prognostic value of clinical tests performed in the immediate post-natal period as predictors of adverse neurological outcomes in HT near-term and term infants with perinatal asphyxia and HIE. Importantly, to investigate which HIE infants would have neurological sequelae, this review only included studies with long-term follow-up, whereby infants were followed until ≥18 months of age. In our opinion, outcomes couldn't be accurately assessed in infants younger than 18 months as neurological sequelae usually manifest at ≥12 months of age, and mental and behavioral disabilities might appear even later (46).

This meta-analysis has several limitations. First, the sample size was small. Second, this review was restricted to articles published in the English language, which may have led to an overestimation or underestimation of prognostic reliability. Finally, heterogeneity was low to moderate, possibly due to differences in thresholds for the index test, the severity of HIE and the design of the studies. Subgroup analyses were not performed to investigate the source of this heterogeneity. Well-designed more extensive studies are required to determine an accurate estimate of the value of clinical tests performed in the immediate post-natal period for predicting in HT infants with HIE.

## Conclusions

This systematic review and meta-analysis provided insight into the prognostic value of clinical tests performed in the immediate post-natal period as predictors of adverse neurological outcomes in HT near-term and term infants with perinatal asphyxia. MRI and neurophysiological tests (aEEG or EEG) were promising predictors of the adverse outcomes, whereas SEPs need high-quality studies to confirm the findings. Given the heterogeneity in the tests' performance, continued follow-up of the children and well-designed large prospective studies are essential to determine whether these benefits are maintained in later childhood.

## Data Availability Statement

All datasets generated for this study are included in the article/Supplementary Material.

## Author Contributions

WL, QY, and ZH performed the screening, extraction of data for included studies, and assessed the quality of study. WD, HW, and YF conducted data extraction of included studies. WL supervises the development of concepts, execution of methodology, analysis, and manuscript writing. All authors reviewed and commented the manuscript.

### Conflict of Interest

The authors declare that the research was conducted in the absence of any commercial or financial relationships that could be construed as a potential conflict of interest.
