# Supplementary material for: Prognostic Value of Clinical Tests in Neonates With Hypoxic-Ischemic Encephalopathy Treated With Therapeutic Hypothermia: A Systematic Review and Meta-Analysis
Source: Front Neurol. 2020 Feb 25;11:133. doi: 10.3389/fneur.2020.00133 (PMC7052385; doi:10.3389/fneur.2020.00133)
Supplement: Supplementary file 1 [file Table_1.pdf]

Supplemental Table 1 Excluded studies and reasons

First Author	Journal and Time	Title	Exclusion reason
Parikh NA	Journal of perinatology 2009	Volumetric and anatomical MRI for hypoxic-ischemic encephalopathy: relationship to hypothermia therapy and neurosensory impairments.	Cannot extract data from the literature
Azzopardi DV	The New England journal of medicine 2009	Moderate hypothermia to treat perinatal asphyxial encephalopathy.	Cannot extract data from the literature
Zhou WH	The Journal of pediatrics 2010	Selective head cooling with mild systemic hypothermia after neonatal hypoxic-ischemic encephalopathy: a multicenter randomized controlled trial in China	Cannot extract data from the literature
Simbruner G	Pediatrics 2010	Systemic hypothermia after neonatal encephalopathy: outcomes of neo.nEURO.network RCT.	Cannot extract data from the literature
Alderliesten T	Radiology 2011	MR imaging and outcome of term neonates with perinatal asphyxia: value of diffusion-weighted MR imaging and ^{1}H MR spectroscopy.	Cannot extract data from the literature
Robertson NJ	Trials 2011	Pilot randomized trial of therapeutic hypothermia with serial cranial ultrasound and 18-22 month follow-up for neonatal encephalopathy in a low resource hospital setting in Uganda: study protocol.	Cannot extract data from the literature
Jacobs SE	Archives of pediatrics & adolescent medicine 2011	Whole-body hypothermia for term and near-term newborns with hypoxic-ischemic encephalopathy: a randomized controlled trial.	Cannot extract data from the literature
Guillet R	Pediatric research 2012	Seven- to eight-year follow-up of the CoolCap trial of head cooling for neonatal encephalopathy.	Cannot extract data from the literature
Roka A	Acta paediatrica (Oslo,	Serum S100B and neuron-specific enolase levels in	Cannot extract data

	Norway : 1992) 2012	normothermic and hypothermic infants after perinatal asphyxia.	from the literature
Cheong JL	Archives of pediatrics & adolescent medicine 2012	Prognostic utility of magnetic resonance imaging in neonatal hypoxic-ischemic encephalopathy: substudy of a randomized trial.	Cannot extract data from the literature
Kato T	Brain & development 2014	A new electroencephalogram classification with reduced recording time in asphyxiated term infants.	Cannot extract data from the literature
Yamamoto Y	Pediatrics international 2015	Calcium concentration in hypoxic-ischemic encephalopathy during hypothermia.	Cannot extract data from the literature
Elstad M	Resuscitation 2016	Heart rate response to therapeutic hypothermia in infants with hypoxic-ischaemic encephalopathy.	Cannot extract data from the literature
Ahearne CE	Developmental neuroscience 2017	Cord Blood IL-16 Is Associated with 3-Year Neurodevelopmental Outcomes in Perinatal Asphyxia and Hypoxic-Ischaemic Encephalopathy.	Cannot extract data from the literature
Thayyil S	Trials 2017	Hypothermia for encephalopathy in low and middle-income countries (HELIX): study protocol for a randomised controlled trial.	Cannot extract data from the literature
Dunne JM	Archives of disease in childhood 2017	Automated electroencephalographic discontinuity in cooled newborns predicts cerebral MRI and neurodevelopmental outcome.	Cannot extract data from the literature
Basu SK	Archives of disease in childhood 2017	Hyperglycaemia in infants with hypoxic-ischaemic encephalopathy is associated with improved outcomes after therapeutic hypothermia: a post hoc analysis of the CoolCap Study.	Cannot extract data from the literature
Jain SV	Early human development 2017	Cerebral regional oxygen saturation trends in infants with hypoxic-ischemic encephalopathy.	Cannot extract data from the literature

Gerner GJ	Journal of perinatology 2018	Transfontanellar duplex brain ultrasonography resistive indices as a prognostic tool in neonatal hypoxic-ischemic encephalopathy before and after treatment with therapeutic hypothermia.	Cannot extract data from the literature
Suppiej A	Clinical neurophysiology 2018	Bilateral loss of cortical SEPs predict severe MRI lesions in neonatal hypoxic ischemic encephalopathy treated with hypothermia.	Cannot extract data from the literature
Weeke LC;	The Journal of pediatrics 2018	A Novel Magnetic Resonance Imaging Score Predicts Neurodevelopmental Outcome After Perinatal Asphyxia and Therapeutic Hypothermia.	Cannot extract data from the literature
Montaldo P	Resuscitation 2018	Electrocardiographic and echocardiographic changes during therapeutic hypothermia in encephalopathic infants with long-term adverse outcome.	Cannot extract data from the literature
Debillon T	BMC pediatrics 2018	LyTONEPAL: long term outcome of neonatal hypoxic encephalopathy in the era of neuroprotective treatment with hypothermia: a French population-based cohort.	Cannot extract data from the literature
Polackova R	Biomedical papers of the Medical Faculty of the University Palacky 2018	Lactate as an early predictor of psychomotor development in neonates with asphyxia receiving therapeutic hypothermia.	Cannot extract data from the literature
Jones R	The journal of maternal-fetal & neonatal medicine 2018	Biochemical and clinical predictors of hypoxic-ischemic encephalopathy after perinatal asphyxia.	Cannot extract data from the literature
Arriaga-Redondo M	Therapeutic hypothermia and temperature management 2019	Lack of Variability in Cerebral Oximetry Tendency in Infants with Severe Hypoxic-Ischemic Encephalopathy Under Hypothermia.	Cannot extract data from the literature

Rao R	American journal of perinatology 2019	Neurodevelopmental Outcomes in Neonates with Mild Hypoxic Ischemic Encephalopathy Treated with Therapeutic Hypothermia.	Cannot extract data from the literature
Lemmers PM	Pediatric research 2013	Cerebral oxygenation and brain activity after perinatal asphyxia: does hypothermia change their prognostic value?	Data can be extracted, but Small sample size does not allow meta-analysis
Kelen D	PloS one 2017	Serum copeptin and neuron specific enolase are markers of neonatal distress and long-term neurodevelopmental outcome.	Data can be extracted, but Small sample size does not allow meta-analysis
Li J	Brain & development 2014	Nucleated red blood cell counts an early predictor of brain injury and 2-year outcome in neonates with hypoxic-ischemic encephalopathy in the era of cooling-based treatment.	Data can be extracted, but Small sample size does not allow meta-analysis
Liu CQ	Zhongguo dang dai er ke za zhi 2010	Effects of selective head cooling with mild hypothermia on serum levels of caspase-3 and IL-18 in neonates with hypoxic-ischemic encephalopathy	Non-English language
Delnard N	Archives de pediatrie 2010	Assessment of a hypothermia protocol implementation for hypoxic-ischemic encephalopathy in term newborns	Non-English language
Blanco D	Anales de pediatria 2011	Neuroprotection with hypothermia in the newborn with hypoxic-ischaemic encephalopathy. Standard guidelines for its clinical application].	Non-English language
Sakurai A	Nihon rinsho 2011	Therapeutic hypothermia	Non-English language
Bekiesińska-	Ginekologia polska 2013	Regression of cystic lesions on brain MRI in a child with	Non-English language

Figatowska M		hypoxic-ischemic encephalopathy treated with selective head cooling	
Zhang P	Zhongguo dang dai er ke za zhi 2013	Research progress in mild hypothermia treatment of neonatal hypoxic-ischemic encephalopathy	Non-English language
Helsmoortel A	Archives de pediatrie 2013	Neonatal therapeutic hypothermia: amplitude-integrated electroencephalography to confirm the indication	Non-English language
Martínez-Biarge M;Blanco D	Anales de pediatria 2014	Follow-up of newborns with hypoxic-ischaemic encephalopathy	Non-English language
Marret S	Archives de pediatrie 2014	Treatment of encephalopathy by hypothermia in the term newborn	Non-English language
Arriaga Redondo M	Anales de pediatria 2016	Stridor in neonates with hypoxic-ischaemic encephalopathy subject to selective cerebral or whole body hypothermia	Non-English language
Nuñez A	Anales de pediatria 2018	Oxidative stress in perinatal asphyxia and hypoxic-ischaemic encephalopathy	Non-English language
Hallberg B	Acta paediatrica (Oslo, Norway : 1992) 2010	The prognostic value of early aEEG in asphyxiated infants undergoing systemic hypothermia treatment.	Follow up < 18 month
Massaro AN	Journal of perinatology 2010	Quantitative analysis of magnetic resonance images and neurological outcome in encephalopathic neonates treated with whole-body hypothermia.	Follow up < 18 month
Bonifacio SL	The Journal of pediatrics 2011	Perinatal events and early magnetic resonance imaging in therapeutic hypothermia.	Uncertain follow-up time
Gucuyener K	Brain & development 2012	Use of amplitude-integrated electroencephalography (aEEG) and near infrared spectroscopy findings in neonates with asphyxia during selective head cooling.	Follow up < 18 month

Ancora G	Brain & developmen 2013	Early predictors of short term neurodevelopmental outcome in asphyxiated cooled infants. A combined brain amplitude integrated electroencephalography and near infrared	Follow up < 18 month
Briatore E	Brain & development 2013	EEG findings in cooled asphyxiated newborns and correlation with site and severity of brain damage.	Uncertain follow-up time
Rollins N	Pediatric neurology 2014	Predictive value of neonatal MRI showing no or minor degrees of brain injury after hypothermia	Follow up < 18 month
Skranes JH	Neonatology 2014	Hypothermia makes cerebral resistance index a poor prognostic tool in encephalopathic newborns.	Uncertain follow-up time
Nanavati T	Pediatrics and neonatology 2015	Can We Predict Functional Outcome in Neonates with Hypoxic Ischemic Encephalopathy by the Combination of Neuroimaging and Electroencephalography?	Uncertain follow-up time
Goeral K	Neonatology 2017	Prediction of Outcome in Neonates with Hypoxic-Ischemic Encephalopathy II: Role of Amplitude-Integrated Electroencephalography and Cerebral Oxygen Saturation Measured by Near-Infrared Spectroscopy.	Uncertain follow-up time
Sijens PE	Magnetic resonance imaging 2017	The prognostic value of proton magnetic resonance spectroscopy in term newborns treated with therapeutic hypothermia following asphyxia.	Follow up < 18 month
Goeral K	Neonatology 2017	Prediction of Outcome in Neonates with Hypoxic-Ischemic Encephalopathy II: Role of Amplitude-Integrated Electroencephalography and Cerebral Oxygen Saturation Measured by Near-Infrared Spectroscopy.	Uncertain follow-up time
Mendler MR	Neonatology 2018	Predictive Value of Thompson-Score for Long-Term Neurological and Cognitive Outcome in Term Newborns with Perinatal Asphyxia and Hypoxic-Ischemic	Follow up < 18 month

		Encephalopathy Undergoing Controlled Hypothermia Treatment.	
Procianoy RS	The journal of maternal-fetal & neonatal medicine 2018	Therapeutic hypothermia for neonatal hypoxic-ischemic encephalopathy: magnetic resonance imaging findings and neurological outcomes in a Brazilian cohort.	Uncertain follow-up time
Dereymaeker A	Pediatrics and neonatology 2019	Automated EEG background analysis to identify neonates with hypoxic-ischemic encephalopathy treated with hypothermia at risk for adverse outcome: A pilot study.	Uncertain follow-up time
